# Seroprevalence of *Strongyloides stercoralis* infection in a South Indian adult population

**DOI:** 10.1371/journal.pntd.0010561

**Published:** 2022-07-20

**Authors:** Saravanan Munisankar, Anuradha Rajamanickam, Suganthi Balasubramanian, Satishwaran Muthusamy, Chandra Kumar Dolla, Pradeep Aravindan Menon, Ponnuraja Chinnayan, Christopher Whalen, Paschaline Gumne, Inderdeep Kaur, Varma Nadimpalli, Akshay Deverakonda, Zhenhao Chen, John David Otto, Tesfalidet Habitegiyorgis, Harish Kandaswamy, Thomas B. Nutman, Subash Babu

**Affiliations:** 1 National Institutes of Health-National Institute for Research in Tuberculosis-International Center for Excellence in Research, Chennai, India; 2 National Institute for Research in Tuberculosis, Chennai, India; 3 Office of Cyber Infrastructure and Computational Biology, National Institute of Allergy and Infectious Diseases, National Institutes of Health, United States of America; 4 Laboratory of Parasitic Diseases, National Institutes of Allergy and Infectious Diseases, National Institutes of Health, Bethesda, Maryland, United States of America; University of Passo Fundo: Universidade de Passo Fundo, BRAZIL

## Abstract

**Background:**

The prevalence of *Strongyloides stercoralis* infection is estimated to be 30–100 million worldwide, although this an underestimate. Most cases remain undiagnosed due to the asymptomatic nature of the infection. We wanted to estimate the seroprevalence of *S*. *stercoralis* infection in a South Indian adult population.

**Methods:**

To this end, we performed community-based screening of 2351 individuals (aged 18–65) in Kanchipuram District of Tamil Nadu between 2013 and 2020. Serological testing for *S*. *stercoralis* was performed using the NIE ELISA.

**Results:**

Our data shows a seroprevalence of 33% (768/2351) for *S*. *stercoralis* infection which had a higher prevalence among males 36% (386/1069) than among females 29.8% (382/1282). Adults aged ≥55 (aOR = 1.65, 95% CI: 1.25–2.18) showed higher adjusted odds of association compared with other age groups. Eosinophil levels (39%) (aOR = 1.43, 95% CI: 1.19–1.74) and hemoglobin levels (24%) (aOR = 1.25, 95% CI: 1.11–1.53) were significantly associated with *S*. *stercoralis* infection. In contrast, low BMI (aOR = 1.15, 95% CI: 0.82–1.61) or the presence of diabetes mellitus (OR = 1.18, 95% CI: 0.83–1.69) was not associated with *S*. *stercoralis* seropositivity.

**Conclusions:**

Our study provides evidence for a very high baseline prevalence of *S*. *stercoralis* infection in South Indian communities and this information could provide realistic and concrete planning of control measures.

## Author summary

Strongyloidiasis is a neglected tropical disease caused by a parasite which is endemic to parts of Latin America, Asia and Africa. Due to its characteristic life cycle, individuals can carry the infection lifelong with few or no symptoms. With the aim of knowing the prevalence of *S*. *stercoralis* at the community level, a population in the rural area of South India was studied using serologic assessments. There was a seroprevalence of *S*. *stercoralis* infection in this study of 33%. This suggests a need to revise control programs in order to avoid continued transmission.

## Introduction

*Strongyloides stercoralis* (*Ss*), a nematode of medical importance has a tropical and subtropical distribution, affecting 30–100 million individuals [1, 2]. *Strongyloides stercoralis* infection (*Ss*I) is often clinically asymptomatic and long lasting due to the parasites’ auto-infective life cycle and their ability to alter or evade the host immune system [3, 4]. People living in tropical countries like India are prone to a wide array of infectious diseases and hence diagnosis of both symptomatic and asymptomatic *Ss*I in such populations is important to prevent life-threatening complications that may arise due to possible co-infections.

Coproparasitological tests are conventionally used to diagnose *S*. *stercoralis*. Most soil transmitted helminths (STH) prevalence studies use faecal egg counting methods such as Kato-Katz, MiniFLOTAC and McMaster´s, despite being complex and relatively less sensitive [2]. A recent systematic review also stated that qPCR technique although shown to be superior in prior reports, has not shown significant sensitivity [5]. Most surveys use serology as a reliable tool for prevalence estimations of *Ss*I [6, 7].

A diverse range of commercial kits and in-house tests using either crude or recombinant antigens have been used in the following techniques–Enzyme-Linked Immunosorbent Assay (ELISA), Indirect Fluorescent Antibody Test (IFAT), Luminex, and Luciferase Immunoprecipitation System (LIPS) for the diagnosis of *Ss*I [8, 9]. These serological assays show sensitivity from 70% to 100% though specificity remains a challenge in endemic regions because of serologic cross-reactivity with other helminths [10]. The 31-kDa NIE recombinant antigen, represents an alternative basis for serological diagnosis, with sensitivities 84–98% and specificities 95–100%, in comparison to the crude antigen-based ELISA [11,12, 13].

In this cross-sectional study, our main objective was to estimate the seroprevalence of *Ss*I based on NIE seroreactivity and to assess potential risk factors for infection acquisition.

## Methods

### Ethics statement

Plasma samples used for this study were collected from individuals who were part of this cross-sectional screening study (NCT01547884) conducted between 2013 and 2020. The study ethical approval was obtained from Institutional Review Boards of the National Institute of Allergy and Infectious Diseases (USA) and National Institute for Research in Tuberculosis (NIRT-IEC-2011 013), Chennai and in adherence to all ethical considerations. A formal written consent was obtained from all adult male and female individuals.

### Study population

This cross-sectional study was performed as a community screening in a South Indian village in Kancheepuram district. Formal written consent was obtained from all adult male and female individuals (aged 18–65 years) before the study procedures. The regions of Sirukalathur and Malayambakkam is primarily supported by agricultural establishments of which the former lies adjacent to a large water body. Additionally, low income and lack of education among the community members have been observed in all screened regions. Poor sanitation is a major drawback in Kollachery and Sikkarayapuram. Medical histories were collected and physical examinations were performed. Household Global Positioning System (GPS) coordinates of all the eligible individuals were also collected. Similar seroprevalence studies were conducted by related groups in closely located urban settings to establish possible variations in occurrence patterns.

### Data variables

The primary outcomes of interest as co-morbidities were diabetes mellitus (DM) or pre-diabetes mellitus (PDM) defined on the basis of HbA1c percentages, using the American Diabetes Association criteria (DM, >6.4%; PDM, 5.7%–6.4%), undernutrition (low body mass index (LBMI)) as described based on the American Heart Association/American College of Cardiology guidelines (LBMI, ≤18.5 kg/m^2^). Additionally, LBMI was confirmed using serum albumin (*<*3.4 g/dl) in all the LBMI individuals (<18.5 kg/m^2^). A complete blood count was done on all samples in a DxH 520 hematology analyzer (Beckman Coulter). Eosinophilia is described as a total eosinophil count of 0.50 × 10^9^ /L or greater [14, 15]. Anemia is defined as a hemoglobin concentration of <13g/dL in males, and <12g/dL in females [16, 17]. In addition, socio-demographic characteristics such as age and sex were analyzed.

### Data collection and management

Paper-based, standardized and structured case reporting forms and e-data capture methods (miForms, REDCap) were used for data collection by trained study staff and the iDatafax clinical data management system was used for secure data management of patient identifiers, demographic, laboratory and clinical data. Maps were made in QGIS 3.10.11; study data were displayed after processing in PostgreSQL and Pentaho. Information from OpenStreetMap and the OpenStreetMap Foundation, was used through an Open Database License. OpenStreetMap data were styled according to guidelines by https://github.com/charlesmillet [18].

### ELISA

*Ss*I was diagnosed by ELISA in plasma to detect the presence of IgG antibodies to *Ss*NIE, derived from *S*. *stercoralis* L3 parasites. The NIE ELISA was performed based on the original protocol by Ravi *et al*. [11] with modifications. The recombinant NIE antigen was coated on 96-well polystyrene plates at a protein concentration of 1.0 μg mL^-1^ in coating buffer and incubated at 4^0^C overnight. The working antigen concentration was determined by prior ‘box titration’ of 10-fold dilutions of a standard positive pool of human plasma versus serial antigen concentrations from 2.0 to 0.062 mg protein per ml. After overnight incubation of antigen, the wells were rinsed and remaining sites blocked by incubation at 37 ^0^ C for 2 h with 150 μl of 5% BSA in PBS and Tween 0.05%. The positive plasma pool was tested in duplicate at 10-fold dilutions ranging from 1:5 to 1:1000 to establish a curve of reactivity. An antibody-negative pool of plasma was also tested at dilutions of 1:5, 1:50, and 1:500. Plasma samples were diluted at 1:500 ratio with dilution buffer containing 1% BSA in PBS and Tween 0.05% and incubated at 37 ^0^ C for 2 h. After the incubation plates were washed with wash buffer and incubated with goat anti-human IgG-conjugated alkaline phosphatase at a 1:500 dilution in dilution buffer for 2 h at 37 ^0^ C. Plates were washed with wash buffer and measured at 405nm (SpectraMax) after developing with *p*-nitrophenol phosphate substrate. The sensitivity and specificity were determined by Receiver Operating Characteristics (ROC) curve analysis using plasma from stool PCR positive *Ss*-infected patients (*n* = 86) and normal healthy controls (stool PCR negative) (*n* = 74). The cut off value was determined as 296 U/ mL^-1^ in correspondence to a sensitivity of 97% and specificity of 95%. Values >296 U/ mL^-1^ are considered as *Ss* positive.

### Statistical analysis

All study data were taken from study database as per the study data handling policy for performing study analyses. All statistical results were based on two-sided tests and the p-value < 0.05 was considered statistically significant. Descriptive analysis was done for basic socio-demographic factors. A logistic regression for dichotomous dependent variables was conducted to obtain odds ratio (OR) for both crude and adjusted regression models. A univariable regression as crude analysis was performed on all commonly reported independent variables and the eligible significant variables were used for inclusion in the adjusted model to further obtain aORs (adjusted ORs) with the respective 95% CI. A binomial test was done to observe binary probability of a single test using Clopper-Pearson exact test with 95% CI. Data were analysed using IBM-SPSS package version 25. REDCap electronic data capture tools were used to collect and manage data, hosted at National Institute for Research in Tuberculosis–International Centre for Excellence in Research (ICMR-NIRT-NIH-ICER), Chennai, which provides 1) an intuitive interface for validated data capture; 2) audit trails for tracking data manipulation and export procedures; 3) automated export procedures for seamless data downloads to common statistical packages; and 4) procedures for data integration and interoperability with external sources [19, 20].

## Results

### Sociodemographic and clinical characteristics

A total of 2351 adults were recruited in this study between 2013 and 2020, of which 768 adults (33%) were seropositive using the NIE ELISA (Table 1). The median age of *Ss* infected adults was 40 years (interquartile range [IQR] 31–50). The prevalence of female *Ss*I positive subjects of the total screened in all villages is 29.8% and male subjects is 36.1% (Table 1) (S1 Fig). The non-communicable outcome of interest as co-morbidity was either obesity (BMI ≥25 kg/m^2^) 30.9% (319/1032) or overweight (BMI 23–24.9 kg/m^2^) 33.5% (144/430), and LBMI (BMI <18.5 kg/m^2^) was 36% (69/192). The prevalence of DM 33.5% (163/459) or PDM 32.4% (255/788) was classified based on HbA1c levels. Being male increased the odds of having *Ss*I 36.1% (OR = 1.33, 95% CI: 1.12–1.58). Age ≥55 [(OR = 1.68, 95% CI: 1.29–2.18) (aOR = 1.66, 95% CI: 1.23–2.23] was associated with *Ss*I both in unadjusted and adjusted analyses; age groups 35–44 (OR = 1.19, 95% CI: 0.95–1.49), (aOR = 1.21, 95% CI: 0.95–1.55) and 45–54 (OR = 1.26, 95% CI: 0.99–1.59), (aOR = 1.23, 95% CI: 0.95–1.60) were not correlated with increased likelihood of infection (Table 2). Of the total *Ss*I positive individuals, 38.8% (302/778) exhibited higher eosinophil levels. Anemic state defined by haemoglobin level was observed in 24.1% (125/518) of females and 29.7% (95/320) of males. Age wise distribution among all villages given with their respective percentages against the total screened subjects can be found in S2 Fig.

**Table 1:** Socio-demographic and clinical characteristics of *Stongyloides stercoralis* infected South Indian adult population enrolled between 2013 and 2020.

Variable	Total n (%)	*Ss* positive n (%) GM (range)	*Ss* negative n (%) GM (range)
***Total*, *n***	2351 (100)	768 (33)	1583 (67)
***Socio-demographic characteristics-Sex***			
Female	1282 (55)	382 (29.8)	900 (70.2)
Male	1069 (45)	386 (36.1)	683 (63.9)
***Age*, *years (median)***			
18–34 (28)	793 (34)	228 (28.8)	565 (71.2)
35–44 (39)	671 (29)	218 (32.5)	453 (67.5)
45–54 (49)	543 (23)	183 (33.7)	360 (66.3)
≥55 (58)	344 (14)	139 (40.4)	205 (59.6)
***BMI (kg/m*** ^***2***^ ***)***			
Normal (18.5–22.9)	697 (30)	236 (33.9) 20.9 (18.5–22.9)	461 (66.1) 21.0 (18.5–22.9)
Undernourished (<18.5)	192 (8)	69 (36) 16.8 (13.5–18.4)	123 (64) 17.0 (12.3–18.5)
Overweight (23.0–24.9)	430 (18)	144 (33.5) 24.0 (23.0–24.9)	286 (66.5) 24.0 (23–24.9)
Obesity (≥25.0)	1032 (44)	319 (30.9) 28.3 (25–41.2)	713 (69.1) 28.6 (25–43.8)
***HbA1c (%)***			
NDM (≤5.7)	1104 (47)	350 (31.7) 5.3 (3.9–5.7)	754 (68.3) 27.9 (18–34)
PDM (>5.7–≤6.4)	788 (33)	255 (32.4) 6.0 (5.7–6.4)	533 (67.6) 27.9 (18–34)
DM (>6.4)	459 (20)	163 (35.5) 8.0 (6.4–18.4)	296 (64.5) 27.9 (18–34)
***Eosinophil Levels*** (≥0.5 cells×10^9^/L)	778 (100)	302 (38.8) 0.4 (0.03–4.04)	476 (61.2) 0.4 (0.03–4.04)
***Hemoglobin*** Female (<12g/dL)	518 (100)	125 (24.1) 13.1 (4.9–22.1)	393 (75.9) 13.1 (4.9–22.1)
Male (<13g/dL)	320 (100)	95 (29.7) 13.1 (4.9–22.1)	225 (70.3) 13.1 (4.9–20.1)

*Ss* = *Stongyloides stercoralis*, *Ss*I *= Strongyloides stercoralis* infection, BMI = body mass index, HbA1c = glycated hemoglobin, NDM = non diabetes mellitus, PDM = pre diabetes mellitus, DM = diabetes mellitus, and GM = geometric mean.

**Table 2:** Association of clinical co-morbidities with *Strongyloides stercoralis* infected South Indian adult population enrolled between 2013 and 2020.

Variable	*Ss*I OR (95% Cl)	*p*-value	*Ss*I aOR (95% Cl)	*p*-value
***Socio-demographic characteristics-Sex***				
Female	Reference	0.001	Reference	0.031
Male	1.33 (1.12–1.58)		1.21 (1.03–1.42)	
***Age*, *years***				
18–34	Reference	0.002	Reference	0.010
35–44	1.19 (0.95–1.49)		1.21 (0.95–1.55)	
45–54	1.26 (0.99–1.59)		1.23 (0.95–1.60)	
≥55	1.68 (1.29–2.18)		1.66 (1.23–2.23)	
***BMI (kg/m*** ^***2***^ ***)***				
Normal (18.5–22.9)	Reference	0.398	Reference	0.540
Undernutrition (<18.5)	1.09 (0.78–1.53)		1.18 (0.83–1.69)	
Overweight (23.0–24.9)	0.98 (0.76–1.26)		0.97 (0.74–1.28)	
Obesity (≥25.0)	0.87 (0.71–1.07)		0.92 (0.73–1.15)	
***HbA1c (%)***				
NDM (≤5.7)	Reference	0.338	Reference	0.961
PDM (>5.7–≤6.4)	1.02 (0.84–1.25)		0.99 (0.80–1.23)	
DM (>6.4)	1.18 (0.94–1.49)		1.03 (0.79–1.35)	
***Eosinophil Levels*** (≥0.5 cells×10^9^/L)	1.52 (1.27–1.83)	0.000	1.43 (1.19–1.74)	0.000
***Haemoglobin*** Male	Reference	0.002	Reference	0.030
Female (<12g/dL)	1.32 (1.11–1.58)		1.25 (1.11–1.53)	

*Ss*I *= Strongyloides stercoralis* infection, BMI = body mass index, HbA1c = glycated hemoglobin, NDM = non diabetes mellitus, PDM = pre diabetes mellitus, DM = diabetes mellitus, Cl = confidence interval, OR = odds ratio, aOR = adjusted odds ratio.

### *Strongyloides stercoralis* geographic distribution

**Fig 1:**
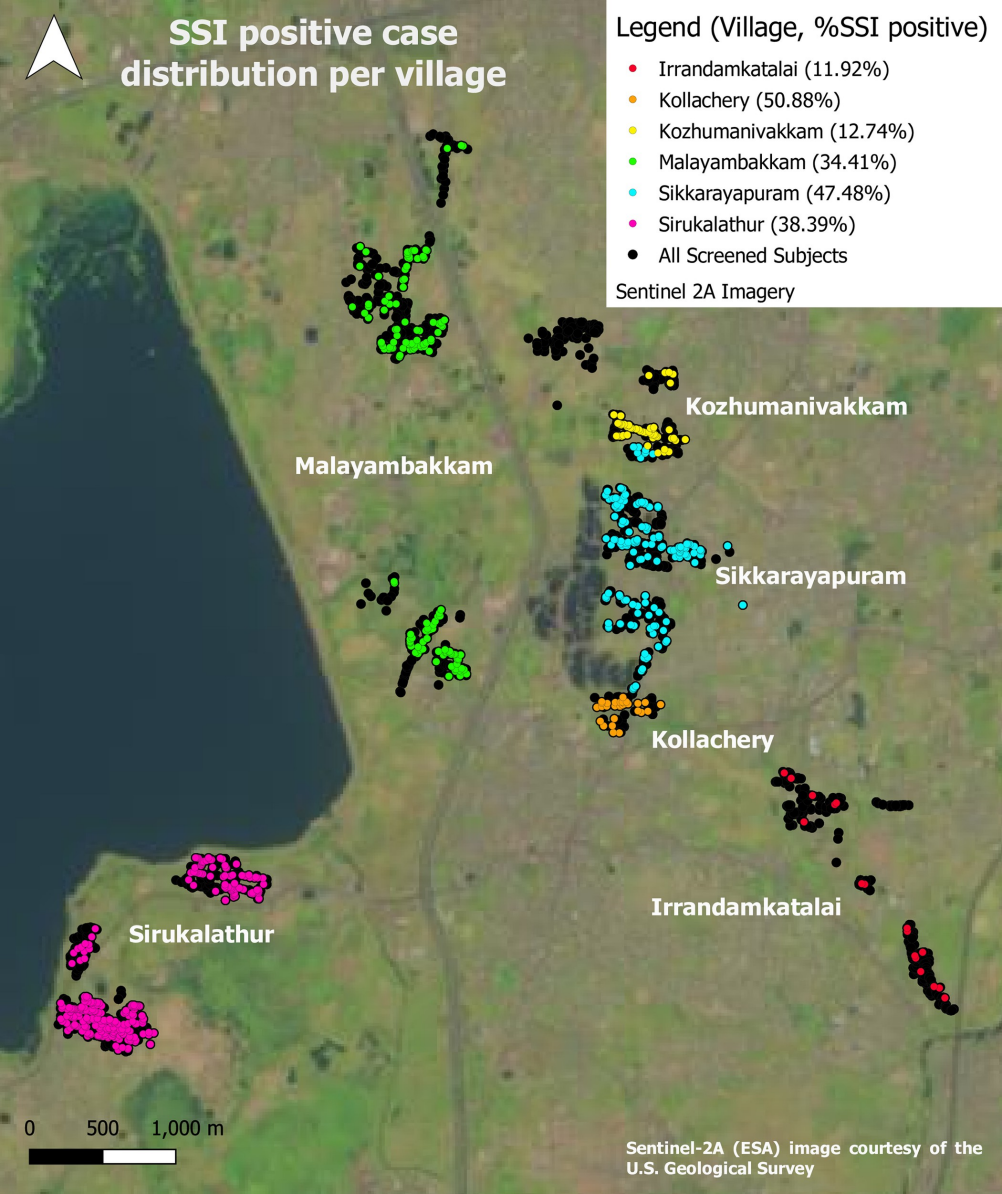
Distribution of *Strongyloides stercoralis* by study site. Map made in QGIS 3.10.11, study data displayed after processing in PostgreSQL and Pentaho. Contains information from OpenStreetMap and OpenStreetMap Foundation, which is made available under the Open Database License. OpenStreetMap data styled according to guidelines by https://github.com/charlesmillet.

Our seroprevalence study was conducted in six villages of Kancheepuram district in South India. The following prevalence rates was observed in the villages–Kollacherry (50.88%; 29/57), Sikkarayapuram (47.48%; 179/377), Sirukalathur (38.39%; 334/870), Malayambakkam (34.41%; 149/433), Kozhumannivakham (12.74%; 59/463) and Irandamkattalai (11.92%; 18/151). The map (Fig 1) depicts the prevalence of *S*. *stercoralis* in each village surveyed and the size of *S*. *stercoralis* infection clusters taken at 5 km radius (S3 Fig). Households with more than one infected person were identified in Sirukalathur (17.4%), Malayambakkam (12.7%), Kozhumannivakham (11.8%), and Sikkarayapuram (11.7%) (4 of the 6 villages surveyed).

### Prevalence of eosinophilia and anemia with *Ss*I in individual villages

Of the 6 villages screened, Kollacherry (50.8%) showed the highest percentage of positivity for *Ss*I followed by Sikkarayapuram (47.4%). The occurrence of eosinophilia was maximum in Irandamkattalai (46.3%). Though screened numbers were minimum, Kollacherry (26.3%) had the highest percentage of positivity for *Ss*I in combination with eosinophilia (Table 3). Additionally, Kollacherry had the highest number of cases of *Ss*I and anemia female (24%) and male (21.4%) followed by Sikkarayapuram female (14%) and male (15.3%). (Table 4).

**Table 3:** Prevalence of Eosinophilia and *Stongyloides stercoralis* infection village wise.

Village screened (no)	*Ss*I N (%)	Eosinophilia N (%)	*Ss*I+Eosinophilia N (%)
Irandamkattalai (151)	18 (11.9)	70 (46.3)	14 (9.3)
Kollacherry (57)	29 (50.8)	19 (33.3)	15 (26.3)
Kozhumannivakham (463)	59 (12.7)	166 (35.8)	34 (7.3)
Malayambakkam (433)	149 (34.4)	150 (34.6)	55 (12.7)
Sikkarayapuram (377)	179 (47.4)	73 (19.3)	46 (12.2)
Sirukalathur (870)	334 (38.3)	300 (34.4)	138 (15.8)

*Ss*I = *Stongyloides stercoralis* infection, N = number

**Table 4:** Prevalence of Anemia and *Stongyloides stercoralis* infection.

Village screened (F/M)	*Ss*I F/M N (%)	Anemia F/M N (%)	*Ss*I + Anemia F/M N (%)
Irandamkattalai (93/58)	9 (9.6)/9 (15.5)	40 (43)/32 (55.2)	5 (5.3)/6 (10.3)
Kollacherry (29/28)	10 (34.4)/19 (67.8)	15 (52)/5 (17.8)	7 (24)/6 (21.4)
Kozhumannivakham (259/204)	26 (10)/33 (16.2)	90 (34.7)/57 (27.9)	9 (3.5)/11 (5.4)
Malayambakkam (245/188)	75 (30.6)/74 (39.4)	80 (32.6)/45 (23.9)	13 (5.3)/21 (11.2)
Sikkarayapuram (221/156)	107 (48.4)/72 (46)	96 (43.4)/40 (25.6)	31 (14)/24 (15.3)
Sirukalathur (435/435)	155 (35.6)/179 (41)	196 (45)/83 (19)	60 (13.8)/27 (6.2)

*Ss*I = *Stongyloides stercoralis* infection, N = number, F = female, M = male.

### Prevalence and association of BMI with *Ss*I

Amongst 768 *Ss* infected individuals with BMI measurements, 33.5% (144/430) participants were overweight (BMI 23–24.9 kg/m^2^), 30.9% (319/1032) were obese (BMI ≥ 25 kg/m^2^), and 36% (69/192) were LBMI (Table 1) (S4 Fig). There was no association observed between LBMI and *Ss*I (aOR = 1.18, 95% CI: 0.83–1.69); nor was there an association between being overweight (aOR = 0.97, 95% CI: 0.74–1.28) or obese (aOR = 0.92, 95% CI: 0.73–1.15) and *Ss*I (Table 2).

### Prevalence and association of DM and PDM with *Ss*I

The median HbA1c of DM, and PDM was respectively 5.8% (IQR 5.4–6.2), and 5.7% (IQR 5.4–6.2). The prevalence and classification of diabetes mellitus in *S*. *stercoralis* infected subjects was screened across six villages (S5 Fig). The odds of having DM is (OR = 1.18, 95% CI 0.94–1.49) with *Ss*I. There was no association between DM (aOR = 1.03, 95% CI 0.79–1.35) and PDM (unadjusted OR = 1.02, 95% CI 0.84–1.25; aOR = 0.99, 95% CI 0.80–1.23) with *Ss*I (Table 2).

### Prevalence and association of eosinophilia and anemia with *Ss*I

The median eosinophil counts in *Ss* positive individuals (>0.5 cells × 10^9^ /L (IQR 0.6–1.3)) were higher compared with *Ss* negative individuals (IQR 0.6–1.0). The OR of individuals with eosinophilia and positive for *Ss* was 1.52 (95% CI: 1.27–1.83) which remained significant after adjusting with possible confounders-aOR 1.43 (95% CI: 1.19–1.74). Anemia was more predominant in *Ss* positive individuals than *Ss* negative (IQR 8.4–10.5). The OR of seropositive female individuals with anemia was 1.32 (95% CI: 1.11–1.58) and remained significant after adjusting with possible confounders-aOR 1.25 (95% CI: 1.11–1.53) (Table 2).

## Discussion

Strongyloidiasis is characterised by persistent infection, occasional dissemination that can be fatal. The present study shows the overall seroprevalence to be 33% among a South Indian adult population studied between 2013 and 2020. The data was obtained using NIE ELISA as the diagnostic technique. A limited understanding of *Ss*I epidemiology remains despite the high prevalence of this infection [21, 22]. Seasonal flooding might determine the survival of *S*. *stercoralis* larvae in areas close to water bodies [23, 24]. We found a positive *Ss*I risk in Sirukalathur (38.39%) and a region of Malayambakkam (34.41%) which are located near water bodies and is also possible that distance to the water capture and other features related to socio-economic factors and human activity could be risk factors.

The regions in our study namely Kollachery (50.88%), and Sikkarayapuram (47.48%) are poor rural areas with higher infection rates, a finding possibly explained by poor sanitation practices. We see similar seroprevalence study conducted by Cimino et al. (2020) [25], in Argentina and Bolivia with 2803 human serum samples analysed by NIE-ELISA, showing 19.6% as positive rate. A comprehensive review on the distribution of *S*. *stercoralis* infection is presented based on community, hospital, refugee and immigrant surveys [26]. We also recorded more than one household contact in 4 of the 6 villages screened, with the highest percentage in Sirukalathur (17.4%) and Malayambakkam (12.7%). Both these villages are agricultural land located close to a large water body. Unorganised housing and sanitation practices could be the major reason for the spread even within a household. A lack of education among the older population could also be a cause of minimal awareness with regards to the spread.

Countries endemic to *Ss*I, such as Brazil and Thailand, have used various methods to assess the positivity rates of the infection. Community based studies in Brazil and Thailand recorded a positivity of 37.4% using the Baermann method and 23.7% using the Koga agar plate culture method respectively. Low sensitivity diagnostic methods have shown a total of 43.5% positivity in 46 African countries. A lower positivity rate of 19.1% was recorded in the United States of America, two-thirds of them being refugees and immigrants. South-east Asia and Western Pacific region have comparatively lower percentage of infection [Australia (13.6%), Japan (12.7%) and India (12.7%)] [27]. However, data cannot be considered conclusive due to low sensitivity methods in diagnosis.

Our results show the prevalence of *Ss*I which had a higher prevalence among male and showed significant association with *Ss*I in both unadjusted and adjusted odds ratio keeping the female population as reference. Some researchers reported that the prevalence of *Ss*I increases with age, but there is no clear explanation. Lindo *et al*., [28] state that their data are similar with most previous studies, which suggest that exposure to parasitic infection, may not be dependent on age or sex. In contrast, our study shows the prevalence of *Ss*I being associated with age ≥55 significantly (p<0.002).

It has been suggested that eosinophils, with their pro and anti-inflammatory properties, play an important role in the innate immune response in parasitic infections. Elevated levels of eosinophil granule proteins in serum was noted in *Ss* infection, suggesting a possible role in tissue remodelling [29]. We previously demonstrated the presence and persistence of plasma levels of eosinophil granular proteins in association to *Ss*I [30]. Eosinophilia has also been described as a potential marker in the diagnosis of *S*.*stercoralis* with 93.5% sensitivity in a study conducted in Mediterranean coast of Spain [31]. On the contrary, only 25% of the seropositive population in a Canadian refugee group was reported with eosinophilia [32]. Our data reveals among the seropositive individuals, 39% had eosinophilia. Irandamkattalai had the highest percentage of eosinophilia (46.3%) while Kollacherry showed the highest percentage for *Ss*I and eosinophilia (26.3%).

We tried to identify several variables as risk factors associated with *Ss*I. Parasitic infections are an important cause of nutritional and energetic stress. Certainly malnutrition and *Ss*I frequently coexist, but the resources for studying the association are limited [33]. Common nutritional indicators such as being underweight, wasting and stunting in children are surrogate indicators of overall well-being, reflecting the burden of infectious diseases [34]. Manifestations of malnutrition are often observed as anemia, micronutrient deficiencies and anthropometric measurements. Our study shows the prevalence of LBMI to be 36% of the total *Ss*I individuals. Nutritional stress resulting from parasitic infection causes hookworm-associated iron deficiency anemia [35]. Our study showed an association of anemia with *Ss* infection particularly in villages close to water bodies, the highest being in Kollacherry in both female (24%) and male (17.8%). However this cannot be conclusive as anemia can also be caused by malnutrition, given the socio- economic conditions of the village.

Diabetic individuals in a *Strongyloides*-endemic regions showed an increased risk of infection [36, 37]. In contrast, a study in Australia found that DM was inversely associated with *Ss*I infection [38]. The relationship between DM and strongyloidiasis has not been definitive. Our data shows that DM is not associated with *Ss*I.

In conclusion, our study represents a clear risk map of *S*. *stercoralis* in a highly endemic setting. We do acknowledge that non-specific cross-reactivities cannot be completely ruled out and is a limitation of our study. Nevertheless, based on these data, the number of infected individuals can be quantified, which allows for realistic and concrete planning of control measures. The predictive map generated can be useful in identifying areas for the generation of additional baseline data, detecting hotspots of infection, planning and prioritizing areas for control interventions including regular administration of anthelminthic (ivermectin) to risk groups, inculcating good hygiene practices, creating awareness among public and by focusing on improving sanitation standards in tropical countries endemic to other infections. This may help bring down the high seroprevalence and further screening studies might help in understanding any association between *Ss*I and other clinical, demographic characteristics.

## Supporting information

S1 Fig Distribution of female and male positive subjects. Distribution of female and male subjects and the percentage of *S*. *stercoralis* infected subjects screened across six villages.
(TIF)

S2 Fig Age wise distribution of *S*. *stercoralis* infected subjects. Age wise distribution of *S*. *stercoralis* infected subjects screened across six villages.
(TIF)

S3 Fig Radius of villages screened for prevalence of *S*. *stercoralis* infection. Prevalence of *S*. *stercoralis* infection screened in six villages of Kancheepuram district with 5 km radius from the center point.
(TIF)

S4 Fig Prevalence of BMI. Prevalence and classification of BMI in *S*. *stercoralis* infected subjects screened across six villages.
(TIF)

S5 Fig Prevalence of diabetes. Prevalence and classification of diabetes mellitus in *S*. *stercoralis* infected subjects screened across six villages.
(TIF)
