# Supplementary figures and images for: Seroprevalence of Strongyloides stercoralis infection in a South Indian adult population
Source: PLoS Negl Trop Dis. 2022 Jul 20;16(7):e0010561. doi: 10.1371/journal.pntd.0010561 (PMC9299326; doi:10.1371/journal.pntd.0010561)

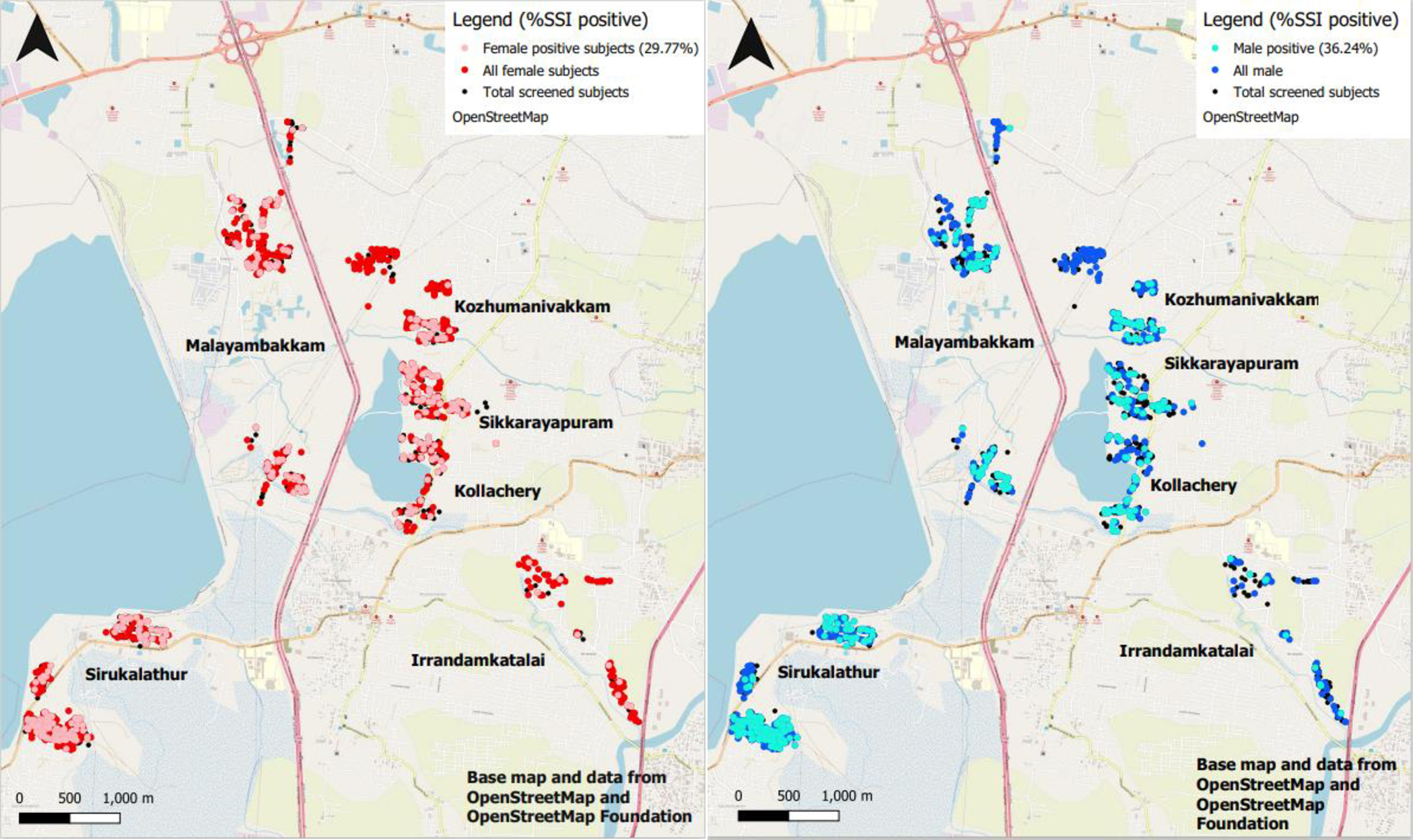

Supplement: S1 Fig — Distribution of female and male subjects and the percentage of S. stercoralis infected subjects screened across six villages. (TIF) [file pntd.0010561.s001.tif]

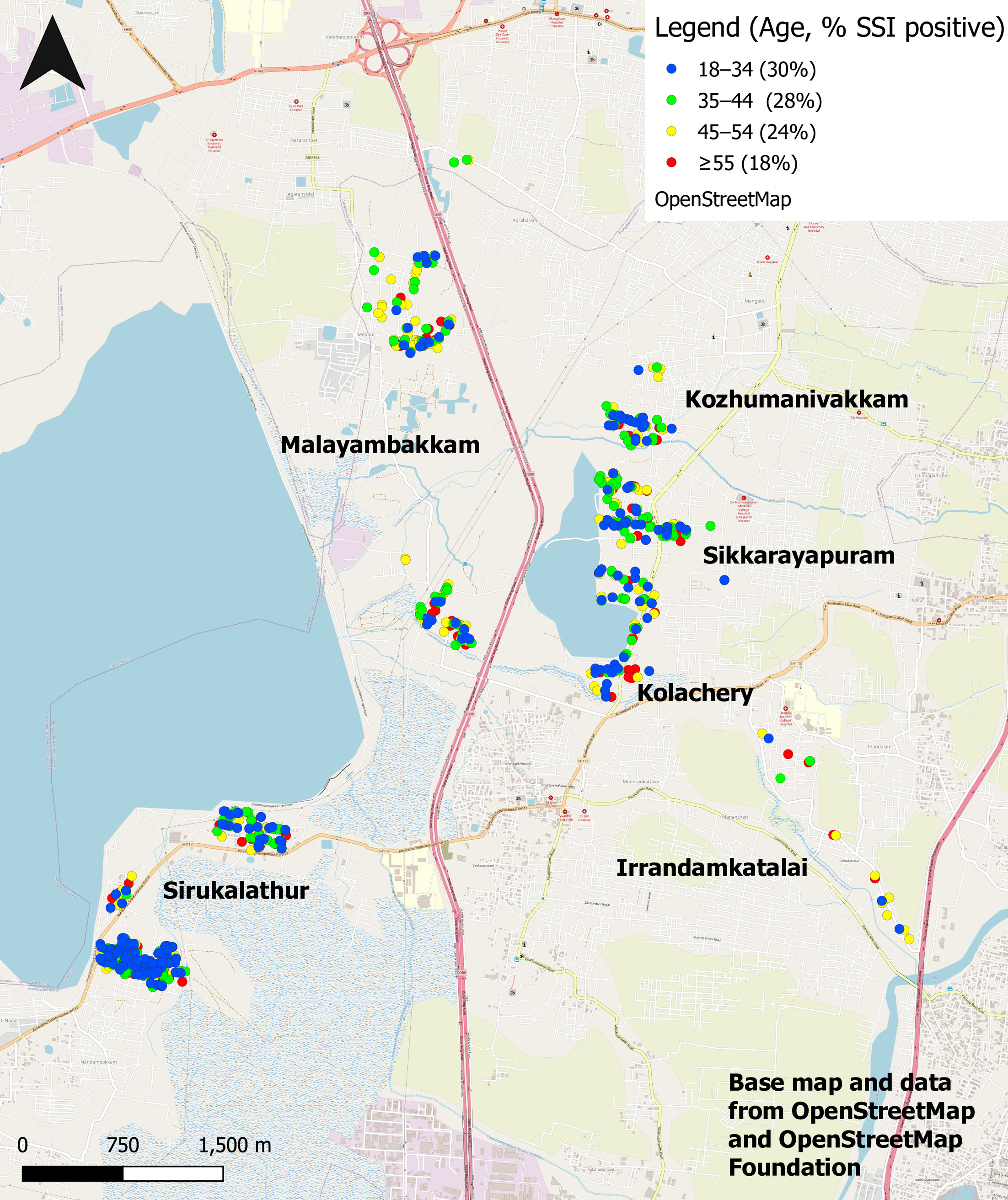

Supplement: S2 Fig — Age wise distribution of S. stercoralis infected subjects screened across six villages. (TIF) [file pntd.0010561.s002.tif]

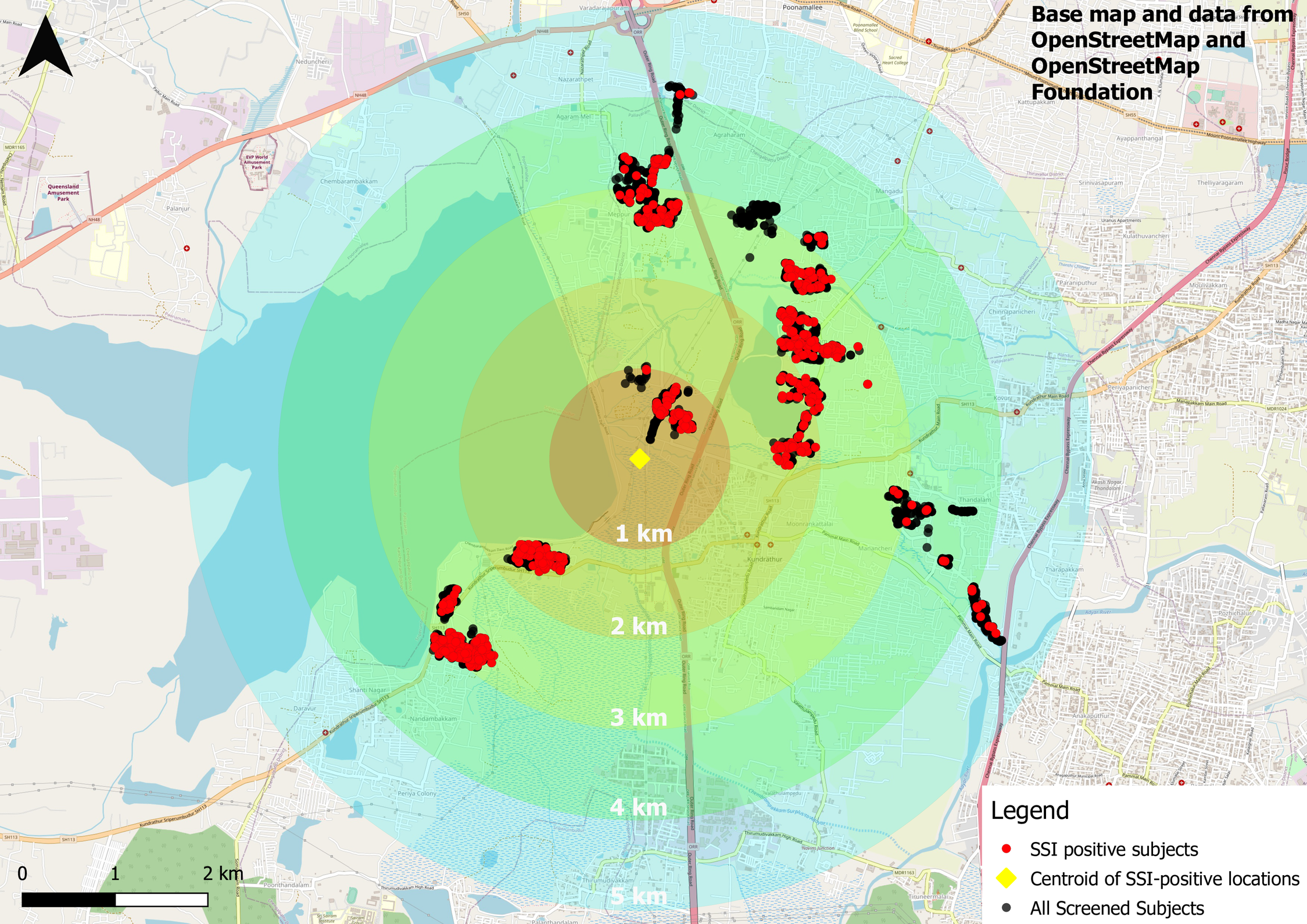

Supplement: S3 Fig — Prevalence of S. stercoralis infection screened in six villages of Kancheepuram district with 5 km radius from the center point. (TIF) [file pntd.0010561.s003.tif]

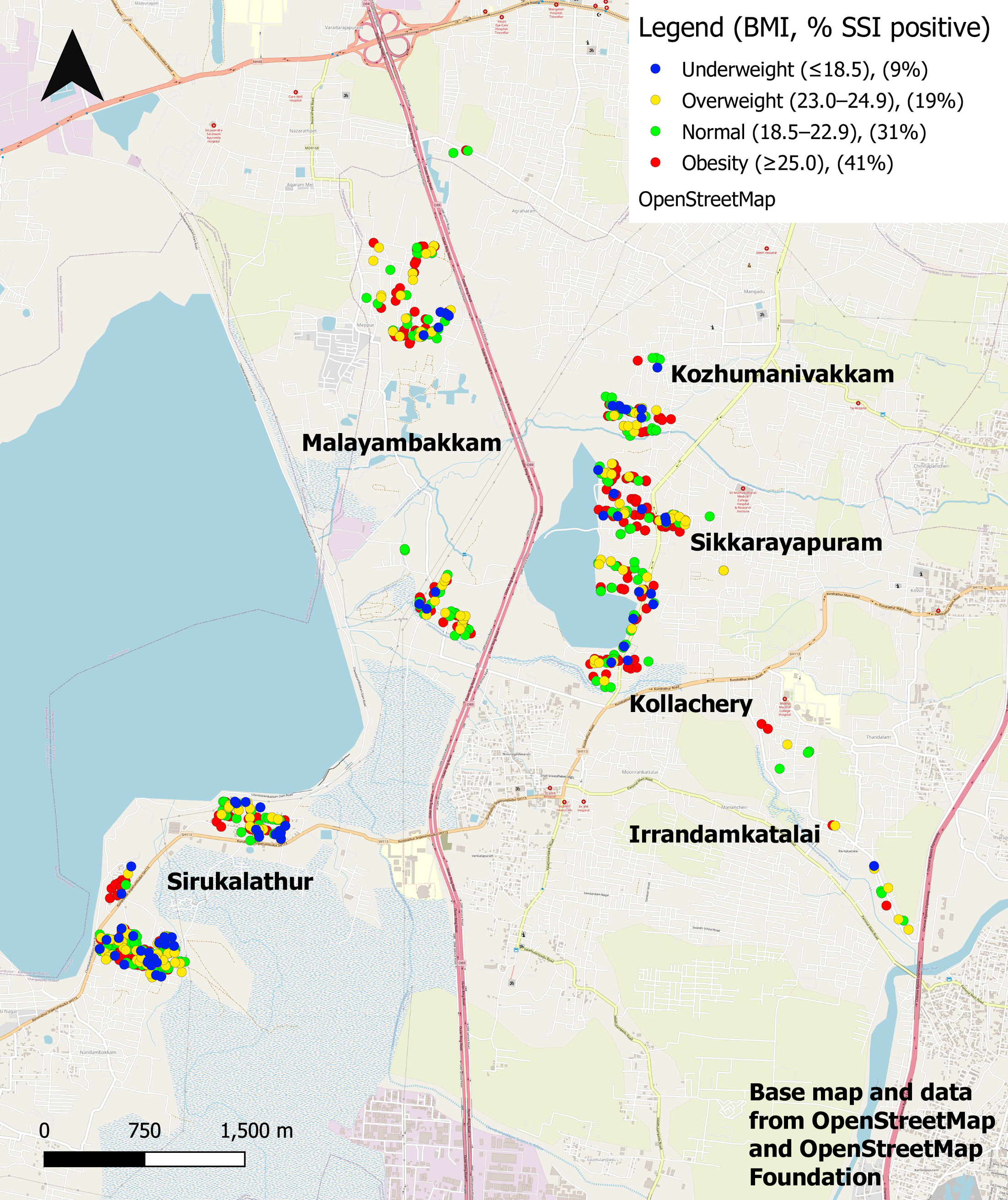

Supplement: S4 Fig — Prevalence and classification of BMI in S. stercoralis infected subjects screened across six villages. (TIF) [file pntd.0010561.s004.tif]

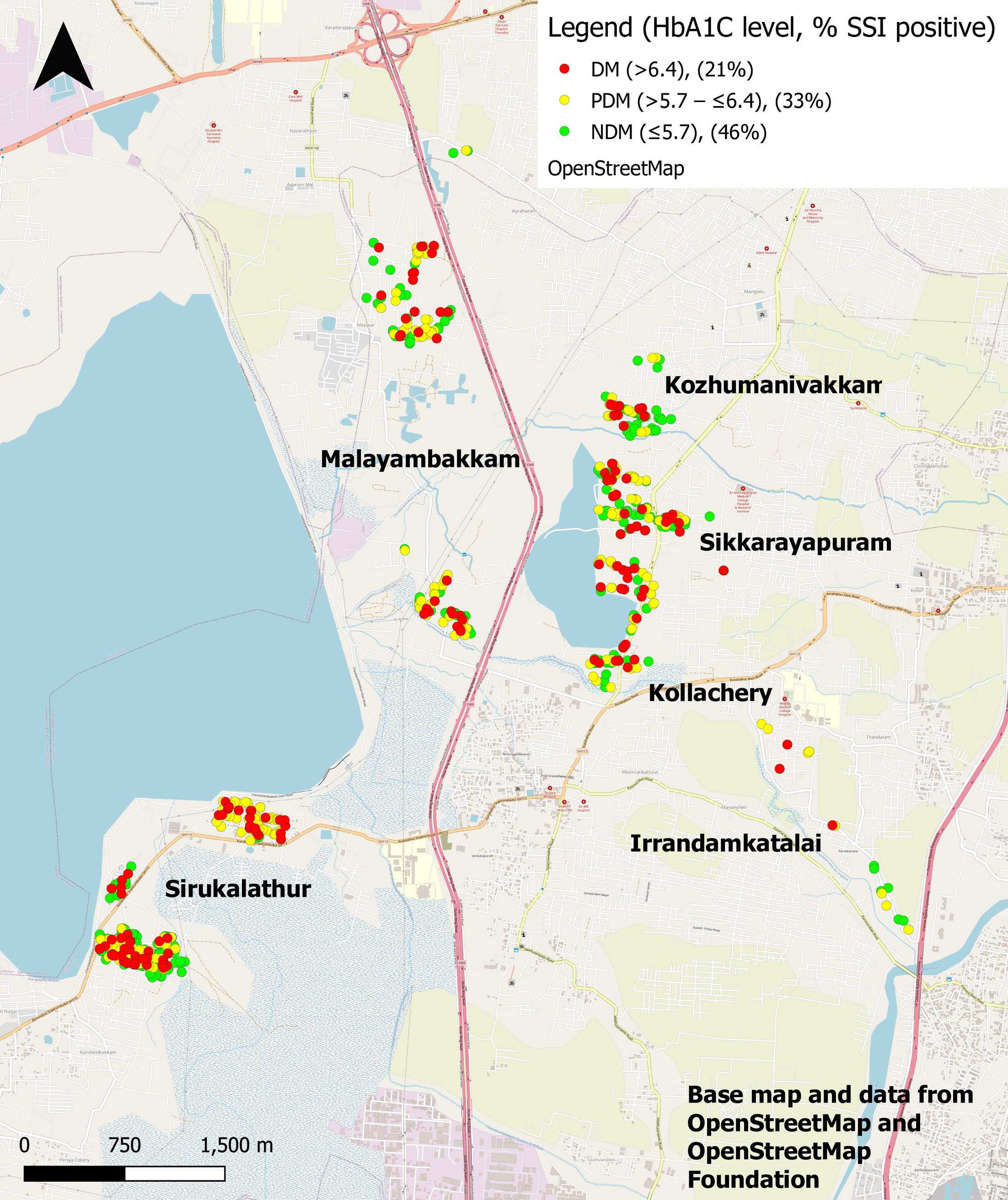

Supplement: S5 Fig — Prevalence and classification of diabetes mellitus in S. stercoralis infected subjects screened across six villages. (TIF) [file pntd.0010561.s005.tif]
